# P-54. Outcomes of patients with MRSA bacteremia treated with vancomycin vs non-vancomycin agents: A tertiary care hospital retrospective review

**DOI:** 10.1093/ofid/ofaf695.283

**Published:** 2026-01-11

**Authors:** Renato Bobadilla Leon, Shira Abeles, Nancy Law, Nina Haste, Victor Chen, Christopher Baladad

**Affiliations:** UCSD, San Diego, CA; University of California San Diego, San Diego, California; University of California, San Diego, La Jolla, California; University of California San Diego, San Diego, California; UCSD, San Diego, CA; UCSD, San Diego, CA

## Abstract

**Background:**

MRSA bloodstream infections have a higher inpatient mortality rate compared to other bloodstream infections, constituting a significant burden to patient outcomes. Vancomycin remains the backbone treatment for MRSA bacteremia despite some studies having shown superiority or non-inferiority of other anti-MRSA agents such as daptomycin. There remains uncertainty in treating MRSA bacteremia patients as to whether vancomycin portents better outcomes compared to non-Vancomycin agents.

**Figure:**
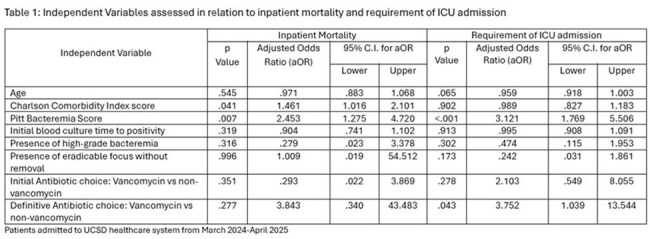

**Methods:**

A retrospective cohort of patients with MRSA bacteremia admitted from March 2024 to April 2025 were extracted from our institution medical records. We compared various metrics associated with care and clinical outcomes for patients with a diagnosis of MRSA bacteremia who were either treated with Vancomycin (VP) vs treated with Non-Vancomycin Agents (NVP). Primary endpoint was all cause inpatient mortality. A multivariate logistic regression was conducted. Secondary end points included: requirement of ICU admission, vasopressor use, development of acute kidney injury that required hemodialysis (AKI-HD), hospitalization days since the first MRSA positive blood culture (MRSA-LoS) and time to clearance of blood cultures (TTC). To compare MRSA-LoS and TTC, a multivariate linear regression was conducted.

**Results:**

A total of 88 hospitalizations during which a patient had MRSA bacteremia met the eligibility criteria. No statistically significant difference in inpatient mortality was noted (VP: 12% vs. NVP: 5.3%; p = 0.277), but Charlson Comorbidity Index (CCI) (aOR 1.46, p=0.041) and Pitt Bacteremia Score (PBS) (aOR 2.45; p=0.007) were predictors of inpatient mortality. Treatment with Vancomycin (aOR 3.75, p=0.043) and PBS (aOR 3.12, p< 0.001) were statistically significant predictors of ICU admission (Table 1). There was no statistically significant difference in vasopressor use (p=0.607), development of AKI-HD (p=0.401), MRSA-LoS (p=0.318) or TTC (p=0.906) between VP vs NVP.

**Conclusion:**

Antibiotic choice vancomycin vs non-vancomycin as definitive therapy was not associated with a clear difference in inpatient mortality for the treatment of MRSA bacteremia in this retrospective analysis. Conversely, receiving Vancomycin as definitive therapy was associated with ICU admission.

**Disclosures:**

All Authors: No reported disclosures

